# Evaluating the Effects of Viruses on Eastern Indigo Snakes (*Drymarchon couperi*) with Gastric Cryptosporidiosis

**DOI:** 10.3390/v16091496

**Published:** 2024-09-21

**Authors:** James E. Bogan, Robert J. Ossiboff, April L. Childress, James F. X. Wellehan, Alexandra K. Mason

**Affiliations:** 1 Central Florida Zoo & Botanical Gardens’ Orianne Center for Indigo Conservation, 30931 Brantley Branch Road, Eustis, FL 32736, USA; 2 Department of Comparative, Diagnostic, and Population Medicine, College of Veterinary Medicine, University of Florida, Gainesville, FL 32610, USA

**Keywords:** adenovirus, coinfection, cryptosporidiosis, *Cryptosporidium serpentis*, *Drymarchon couperi*, reovirus

## Abstract

A breeding colony of wild-origin eastern indigo snakes (EISs, *Drymarchon couperi*) that is part of a reintroduction program has been impacted by gastric cryptosporidiosis. Gastric cryptosporidiosis is an insidious disease of squamates caused by an apicomplexan protozoan, *Cryptosporidium serpentis*. Viral coinfections have been implicated as possible immunosuppressant agents that allow for disease progression and both adenovirus and reovirus have been implicated in allowing for the progression of gastric cryptosporidiosis during coinfection in other snake species. Molecular (PCR) screening for adenoviruses and reoviruses was performed for both *C. serpentis*-positive and *C. serpentis*-negative EIS within the breeding colony. No reoviruses were detected in the collection. Adenoviruses were present in 11/68 (16.2%) EISs evaluated, and there was no significant difference between *C. serpentis*-positive and *C. serpentis*-negative EISs (*p* = 0.196). There was no significant difference in adenovirus status between *C. serpentis*-positive EISs’ lifespan (*p* = 0.191) or survival rates (*p* = 0.823). These findings suggest that the presence of the adenoviruses found in this study does not contribute to the formation or progression of gastric cryptosporidiosis in EISs.

## 1. Introduction

Eastern indigo snakes (EIS, *Drymarchon couperi*) are large diurnal colubrids native to the southeastern USA and are federally listed as a Threatened Species by the Endangered Species Act [1]. While species recovery and reintroduction efforts are in place, infectious disease has impacted breeding initiatives, threatening conservation efforts. Gastric cryptosporidiosis is an insidious disease of squamates characterized by mucosal proliferation that is capable of progressing to luminal occlusion of the stomach and associated clinical signs of anorexia, regurgitation, and subsequent death due to malnutrition and/or sepsis [2]. By far the most common causative agent for gastric cryptosporidiosis in squamates (snakes and lizards) is the apicomplexan protozoan, *Cryptosporidium serpentis.* This protozoan parasite is resistant to both common medications and environmental disinfectants, making both disease prevention and management difficult.

Given the insidious and practically uncurable nature of this disease, it is necessary not only to prevent exposure to *C. serpentis* but also to limit factors that may influence rates of infection or disease severity. One such factor that is poorly characterized is the role that viral coinfections may play in modulating cryptosporidiosis. Reports of snakes with concurrent gastric cryptosporidiosis and systemic viral infections have been previously reported. Five wild-caught rough green snakes (*Opheodrys aestivus*) with gastric cryptosporidiosis were reported to be coinfected with a reovirus [3]. A group of bull snakes (*Pituophis catenifer*) were found to be infected with an unspeciated *Cryptosporidium* and had adenovirus-like particles that were revealed via electron microscopy [4]. Additionally, a single case report of a pet corn snake (*Elaphe guttata guttata*) with gastric cryptosporidiosis demonstrated a coinfection with Snake adenovirus 1 [5].

Since immunocompetency is a primary factor in determining a successful outcome when managing cryptosporidiosis [6], investigating the potential roles of viral coinfections may play in squamate gastric cryptosporidiosis is needed. The focus of this study was to screen for the presence of reoviruses and adenoviruses in EISs with gastric cryptosporidiosis and to look for correlations with clinical outcomes.

## 2. Materials and Methods

### 2.1. Sample Collection and Processing

As part of routine infectious disease surveillance, cloacal swabs collected from EIS housed at the Central Florida Zoo & Botanical Gardens’ Orianne Center for Indigo Conservation from December 2015 through July 2023 were submitted for *C. serpentis*-specific probe hybridization qPCR (partially reported in [7]). Of the stored samples, a single sample was evaluated for adenovirus from 68 individual EISs. Nucleic acids (extracted as described in [7]) were screened for the presence of adenovirus by nested consensus qualitative PCR. Briefly, degenerate primers designed to broadly detect adenoviral DNA polymerase coding sequences were used in two consecutive PCR reactions on EIS samples, as previously described [8]. In round 1, forward primer PolFout (5′-TNMGNGGNGGNMGNTGYTAYCC-3′) and reverse primer PolRout (5′-GTDGCRAANSHNCCRTABARNGMRTT-3′) were used; in the second-round reaction, the round 1 product was subjected to PCR using the forward primer PolFin (5′-GTNTWYGAYATHTGYGGHATGTAYGC-3′) and the reverse primer PolRin (5′-CCANCCBCDRTTRTGNARNGTRA-3′). PCR products were visualized by gel electrophoresis, and amplicons of the appropriate size (~320 base pairs) were gel-extracted and submitted for bidirectional commercial (Genewiz Inc., South Plainfield, NJ, USA) Sanger sequencing. Unfortunately, due to the utilization of reagents focused on DNA extraction in these samples, screening for reoviruses was not possible.

To assess for the presence of reoviral coinfections in EISs with gastric cryptosporidiosis, 29 cloacal samples were collected from *C. serpentis*-positive adult EISs using nylon flocked swabs (Hardy Diagnostics, Santa Maria, CA, USA) in June 2022. Eleven of these EISs also had stored cloacal samples screened for adenovirus, as previously described. Gastric cryptosporidiosis was diagnosed by probe-hybridization qPCR [7] and histologic analysis [2] for all 29 snakes using gastroscopic biopsies collected as previously described [9]. RNA was extracted from the swabs and evaluated for reovirus through nested reverse transcription-PCR (nrt-PCR) targeting the viral RNA polymerase. Briefly, degenerate primers designed to broadly detect mammalian orthoreoviruses and piscine aquareoviruses and previously used to amplify diverse reptile, avian, and mammalian reoviruses [10] were used in two consecutive PCR reactions on EIS samples. In round 1, forward primer 1607F (5′-CARMGNCGNSCHMGHTCHATHATGCC-3′) and reverse primer 2608R (5′-TAVAYRAAVGWCCASMHNGGRTAYTG-3′) were used; in the second-round reaction, the round 1 product was subjected to PCR using the forward primer 2090F (5′-GGBTCMACNGCYACYTCBACYGAGCA-3′) and reverse primer 2334R (5′-CDATGTCRTAHWYCCANCCRAA-3′).

All EISs were housed according to guidelines provided by the Association of Zoos and Aquariums [11].

### 2.2. Statistical Analysis

Categorical data were analyzed with Pearson’s chi-square and Fisher’s exact test. Logistic regression was used to compare data. Analyses were computed using Excel (version 2409, Microsoft 365, Redmond, WA, USA) and Real Statistics add-in software (version 7.9. https://www.real-statistics.com/free-download/real-statistics-resource-pack/ (accessed on 3 December 2021). The statistical significance was set at *p* < 0.05.

For phylogenetic analysis, the predicted homologous regions of nucleotide and amino acid sequences of 59 adenoviral partial DNA polymerases either were downloaded from GenBank or represent novel adenovirus sequences detected in historical non-EIS submissions to the Zoological Medicine Diagnostic Laboratory at the College of Veterinary Medicine, University of Florida (and also submitted to GenBank) screened using the same adenovirus PCR protocol as described for EIS samples. Sequence ambiguities were added if ends were missing, and amino acid sequences were aligned using MAFFT (version 7) [12]. Amino acid alignments were converted to nucleotide alignments using PAL2NAL (version 14) [13]. Bottlenose dolphin adenovirus 2 (GenBank accession no. KR0247101), in the genus *Mastadenovirus*, was designated as the outgroup. Bayesian analyses were performed using MrBayes 3.2.7 [14] on the CIPRES [15] server, with gamma-distributed rate variation, a proportion of invariant sites, and mixed amino acid substitution models [16]. For the Bayesian analyses, four chains were run, each one for 2,000,000 generations: three hot chains and one cold chain. Convergence among different runs was evaluated by calculating the average split deviation using a threshold of 0.02%. Chains were sampled every 100 generations, and the first 25% were discarded during burn-in.

## 3. Results

Eleven of the sixty-eight (16.2%) banked EIS cloacal samples were PCR-positive for adenovirus. Six EIS samples from 2017 had a virus that had 92% nucleotide sequence identity with Snake adenovirus 1 (GenBank # NC009989) and 100% nucleotide sequence identity with viruses from two beaded lizards (*Heloderma horridum*) from Florida, USA, earlier in 2017 that had recently arrived from Texas, USA, as well as a pine snake (*Pituophis melanoleucus*) and an African mole snake (*Pseudaspis cana*) from Missouri, USA. Three EIS samples from 2020 to 2021 had 100% nucleotide sequence identity with Snake adenovirus 1, one EIS sample from 2022 had a sequence that was 98% homologous to Snake adenovirus 1, and one EIS sample from 2017 had mixed barthadenovirus sequence, most likely indicative of the presence of two or more distinct adenoviruses in the sample. Previously unpublished adenovirus sequences from EISs and other reptile hosts were submitted to GenBank under accession numbers PQ066458-PQ066483.

**Figure 1:**
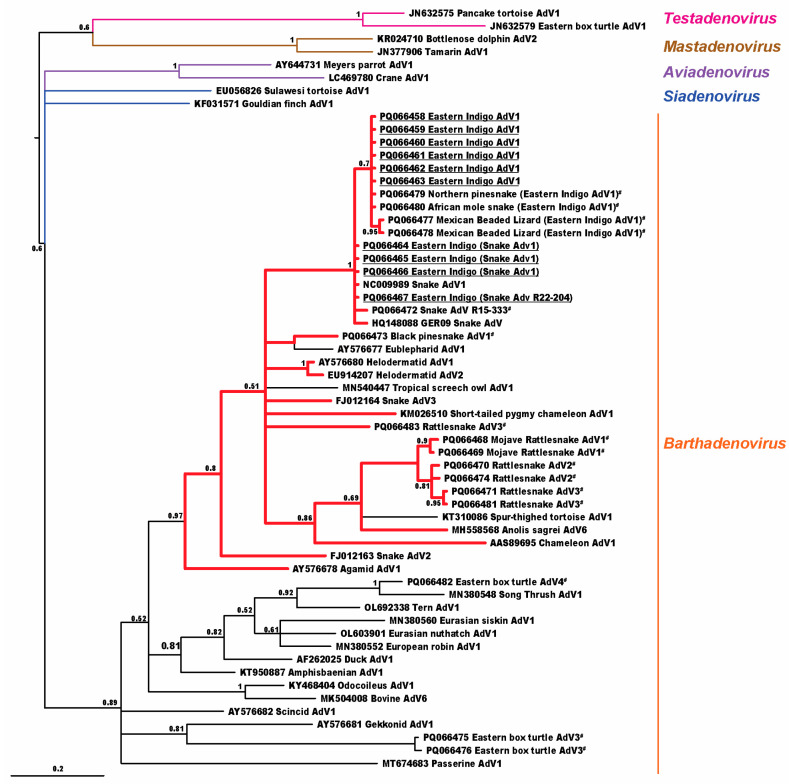
Bayesian analysis cladogram depicting the relationship of adenoviruses detected in eastern indigo snakes (EISs) to other animal hosts. Adenoviral sequences found in EISs are underlined; other adenoviral sequences first reported in this manuscript are also highlighted (^#^). Cladogram branches of viruses detected in toxicoferan hosts are shown in red. Probability values are shown at branch points.

Bayesian phylogenetic analysis (Figure 1) found that the three identified viruses clustered in the genus *Barthadenovirus* (formerly *Atadenovirus*) in a subclade predominantly using squamate hosts in the clade Toxicofera (97% posterior probability).

Thirty-seven of the sixty-eight (54.4%) EISs that were screened for adenovirus also had gastric cryptosporidiosis. There was not a significant difference (Fisher exact *p* = 0.196) in adenovirus status between EISs testing positive for gastric cryptosporidiosis (8/37, 21.6%) when compared to EISs testing negative for gastric cryptosporidiosis (3/31, 9.7%). There was not a significant difference between the different virus types in EISs testing positive for adenovirus when compared to EISs testing positive for gastric cryptosporidiosis (regression *p* = 0.363).

Seventeen of the thirty-seven (46.0%) *C. serpentis*-positive EISs died, with the time of death ranging between 28 and 2637 days from diagnosis. There was not a significant difference in adenoviral status for *C. serpentis*-positive EISs and time of death (regression *p* = 0.191).

Eight of the thirty-seven (21.6%) *C. serpentis*-positive EISs had recovered and tested negative within three years of diagnosis. Negative status was confirmed with histologic and molecular evaluation of gastroscopic biopsies. There was not a significant difference between the adenoviral status for EISs that recovered from *C. serpentis* infection and that of those that did not recover (regression *p* = 0.823).

All 29 EISs with gastric cryptosporidiosis screened by rt-PCR were negative for reovirus.

## 4. Discussion

The adenoviruses found in these snakes had no significant effect on gastric cryptosporidiosis in EIS in this study. None of the EISs with confirmed gastric cryptosporidiosis tested positive for reovirus, so it could not be assessed whether reovirus coinfection may play a role in gastric cryptosporidiosis in EIS.

When comparing adenovirus detection between *C. serpentis*-positive EIS and *C. serpentis*-negative EIS, there was not a significant difference (*p* = 0.196). Although 68 samples represent a relatively high number of tests for comparison in a non-traditional veterinary species, it is still a relatively small number for statistical comparison [17]. This may lead to a type II statistical error. Increasing the number of animals evaluated would likely enable the detection of smaller effects, if present.

Not only was the presence of these adenoviruses not found to be a significant contributor to EIS developing an infection with *C. serpentis*, but it also did not appear to affect the morbidity associated with gastric cryptosporidiosis. The presence of adenovirus did not affect the animal’s ability to clear *C. serpentis*, nor did it shorten the lifespan of *C. serpentis*-positive EISs.

Infection involves complex microbial ecology, and interactions between more than one pathogen and a host may be synergistic or antagonistic. An infectious agent may cause a shift toward an immune pathway that is more or less effective against another potential pathogen. Orf virus infection makes mice less susceptible to avian influenza [18]. Conversely, infection with Gallid alphaherpesvirus 2 makes chickens more susceptible to disease from *Cryptosporidium baileyi* [19].

Data on interactions between adenoviruses and *Cryptosporidium* spp. are limited. In mammals, mastadenoviruses have been documented to evade the immune system, especially cellular immunity, by numerous mechanisms, including MHC expression downregulation, inhibition of TNF function, and downregulation of Fas receptors, preventing apoptosis [20]. In turkeys [21], a siadenovirus has been documented to cause immunosuppression by decreasing the relative proportion of IgM-bearing cells [21]. Further investigation by Suresh and Sharma [21] has shown that adenoviral infection actually causes a relative elevation to CD4^+^ cells in turkeys.

The adenoviral genes known to be most involved in immune evasion are typically found in the E1 and E3 regions near the 5′ and 3′ ends of the genome, respectively [20]. The presence of genes in these areas is much less conserved than in the central core region, and adenoviral genes in these areas are often genus- or even species-specific. There is a lack of knowledge of immunomodulatory function of genes in these regions found in the genus *Barthadenovirus*.

While several reports have documented viral and protozoal coinfections in reptiles, none have examined whether viral coinfections have played a role in the protozoal pathogenesis [3,4,5,22]. Suppression of CD4^+^ T cells has been shown to be a key factor in the persistence of cryptosporidiosis in mammals [23]. Since adenoviral infections may actually increase CD4^+^ T cells, it is plausible that adenoviral coinfection may actually be beneficial in *Cryptosporidium* infections. Since there was not a significant difference in survival of EIS with *C. serpentis* infection between adenovirus-positive and adenovirus-negative snakes, it likely has little effect, if any at all, in EISs.

The adenoviruses detected in EISs were found in a clade using toxicoferan hosts, a squamate clade which includes snakes, iguanids, chameleons, agamids, monitors, helodermatids, and others. The exceptions in this clade were Eublepharid adenovirus 1 from leopard geckos (*Eublepharis macularius*) and Spur-thighed tortoise adenovirus 1 from a Greek tortoise (*Testudo graeca*), which have been found in animals with adenoviral-like inclusions, and Tropical screech owl adenovirus 1, from owl feces, which may represent either infection in the owl or virus passing through from toxicoferan prey. With the exception of Eublepharid adenovirus 1, other atadenoviruses using squamate hosts diverged basal to the Toxicofera, including Gekkonid adenovirus 1, Scincid adenovirus 1, and Amphisbaenian adenovirus 1, which would be expected with barthadenoviral codivergence with squamates.

While all EIS adenoviruses found in this study formed a monophyletic clade, we caution against expecting different types of adenoviruses to behave similarly in terms of disease severity and host range. As an example, Canine adenovirus 1 and Canine adenovirus 2 are both types of the same species, Canine adenovirus A. However, Canine adenovirus 1 often results in fatal hepatitis, whereas Canine adenovirus 2 disease is often mild or subclinical [24]. Adenoviruses evolve relatively slowly, and the 2017 virus seen in this collection was also present at another site in Missouri. The prior presence of the 2017 virus in beaded lizards shows that this virus is at least capable of jumping to/from another non-snake toxicoferan, and managers should be aware of this. Some adenoviruses appear to have larger host ranges than others, and further study to identify factors impacting host range is needed [25].

In 2020 and 2021, the EIS adenoviruses were Snake adenovirus 1, first characterized in 2002 from a corn snake (*Pantherophis guttatus*) isolate [26]. The temporal separation of Snake AdV1 from the 2017 virus is consistent with a later introduction of Snake adenovirus 1 into the collection. The 2022 virus is distinct from but much more similar to Snake adenovirus 1, and further study would be needed to determine whether it is ecologically distinct.

The presence of an organism in a host does not necessarily mean the organism is causing disease [27]. A literature search did not reveal prior studies evaluating whether the presence of barthadenoviruses plays roles in protozoan pathogenesis. Based on the data summarized in this study, viral coinfections with the adenoviruses found in this study do not seem to play a large role in gastric cryptosporidiosis in eastern indigo snakes.

## Data Availability

The original contributions presented in the study are included in the article; further inquiries can be directed to the corresponding author.
